# From Local to Global Dilemmas in Social Networks

**DOI:** 10.1371/journal.pone.0032114

**Published:** 2012-02-21

**Authors:** Flávio L. Pinheiro, Jorge M. Pacheco, Francisco C. Santos

**Affiliations:** 1 Applications of Theoretical Physics Group, Centro de Matemática e Aplicações Fundamentais, Instituto para a Investigação Interdisciplinar da Universidade de Lisboa, Lisboa, Portugal; 2 Departamento de Matemática e Aplicações, Universidade do Minho, Braga, Portugal; 3 Departamento de Engenharia Informática, Instituto Superior Técnico, Universidade Técnica de Lisboa, Lisboa, Portugal; University of Sheffield, United Kingdom

## Abstract

Social networks affect in such a fundamental way the dynamics of the population they support that the global, population-wide behavior that one observes often bears no relation to the individual processes it stems from. Up to now, linking the global networked dynamics to such individual mechanisms has remained elusive. Here we study the evolution of cooperation in networked populations and let individuals interact via a 2-person Prisoner's Dilemma – a characteristic defection dominant social dilemma of cooperation. We show how homogeneous networks transform a Prisoner's Dilemma into a population-wide evolutionary dynamics that promotes the coexistence between cooperators and defectors, while heterogeneous networks promote their coordination. To this end, we define a dynamic variable that allows us to track the self-organization of cooperators when co-evolving with defectors in networked populations. Using the same variable, we show how the global dynamics — and effective dilemma — co-evolves with the motifs of cooperators in the population, the overall emergence of cooperation depending sensitively on this co-evolution.

## Introduction

Dynamical processes involving populations of individuals constitute paradigmatic examples of complex systems. From epidemic outbreaks to opinion formation and behavioral dynamics [1]–[9], the impact of the underlying web of ties in the overall behavior of the population is well known. In this context, Evolutionary Games [10], [11] provide one of the most sophisticated examples of complex dynamics in which the role of the underlying network topology proves ubiquitous. For instance, when cooperation is modeled as a prisoner's dilemma game, cooperation may emerge (or not) depending on how the population is networked [12]–[37].

Up to now, it has been hard to characterize in detail the global dynamics by which local self-regarding actions lead to a collective cooperative scenario, relating it to the network topology. Indeed, most network studies have been focused on the analysis of the evolutionary outcome of cooperation [14] — either by means of the numerical analysis of steady states [12], [16], [18], [19], [24], [25], [29], [32], [34], [35], [38]–[40] or by means of the analytical determination of the conditions for fixation in the population or by means of the determination of positive inclusive fitness effects for particular homogeneous network interaction structures and low intensities of selection [17], [20], [22] — without characterizing the self-organization process by which one of the strategies outcompetes the other. Here we show how networked individuals, engaging in a prisoner's dilemma (**PD**) of cooperation, give rise to a global, population wide, behavioral dynamics which deviates strongly from the original **PD**, depending sensitively on the underlying network of contacts: Homogeneous networks promote a coexistence dynamics between cooperators and defectors — akin to the Chicken or Snowdrift game [11], [38], [41]–[43] — whereas heterogeneous networks, from broad scale to scale-free [4], [44], favor the coordination between them, similar to the Stag-hunt game [45].

To this end we define a time-dependent variable — that we call the average gradient of selection (***AGoS***) — and use it to track the self-organization of cooperators when co-evolving with defectors under network reciprocity. Similar to existing analytical approaches [43], [46], the ***AGoS*** is able to provide a measure of the change in time of the frequency of cooperative traits under selection. The ***AGoS*** can be computed for arbitrary intensity of selection (see Methods), arbitrary population structure and arbitrary game parameterization. We further prove that the global games are not fixed: they change in time, co-evolving with the motifs of cooperators in the population. The evolutionary outcome of such a self-organization process will depend sensitively on this co-evolution, which can be followed using a time-dependent ***AGoS***.

### Dynamical Model

Let us consider pairwise interactions between individuals who can behave either as a *Cooperator* (***C***) or *Defector* (***D***). Whenever cheated by a ***D***, a ***C*** receives a payoff *S* (the sucker's payoff), while the ***D*** receives *T* (temptation to defect). Mutual cooperation provides *R* (reward) to each player, while mutual defection provides *P* (punishment). One obtains the prisoner's dilemma (**PD**) — the most famous metaphor of cooperation — whenever *T>R>P>S*. We formalize the dilemma in terms of a single parameter *B* (benefit) by defining *T = B*>1, *R* = 1, *S* = 1-*B* and *P* = 0. The results remain quantitatively unaltered if one adopts the more popular parameterization *T = b*, *R = b–c*, *S = −c* and *P* = 0.

In the framework of evolutionary game theory, we adopt a stochastic update rule of social learning, where in each time step a random individual *i* imitates the strategy of a randomly selected neighbor *j* with a probability that increases with the fitness difference between them [14], [24], [47]–[50] (see Methods). In the limit of well-mixed populations of size *N* [10], [11], the frequency *j/N* of ***C***s will increase (decrease) in time depending on whether the gradient of selection [51], [52] 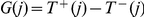 is positive (negative), where 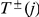 [47] represent the probabilities to increase and decrease the number of ***C***s in the population by one. For the **PD**, 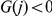 for all *j* and, as a result, cooperation will most probably die out. The same scenario is obtained when *N→∞*, where we recover the scenarios described by the famous replicator dynamics [11], [47]–[50]. The elegance of this result (despite the doomsday scenario for ***C***s) is best appreciated when we realize that the population ends up adopting the Nash-equilibrium of a **PD** game interaction between two individuals: everybody defects. Consequently, there is no difference in the outcome of the game, from an individual or from a (collective) population wide perspective, a feature that, as discussed below, will not remain true in structured populations.

## Results

The previous analysis assumes finite yet structureless populations, a feature which is seldom observed in practice, with strong implications in many natural phenomena. A homogeneous network of size *N* represents the simplest case of a structured population, where all individuals engage in the same number of games *k* with their first neighbors, also imitating their behavior. Let us consider a homogeneous random network — also called a regular random graph — in which all links are randomly connected, while all nodes have each the same number of links [4], [53], [54]. In this case, individuals with the same strategy no longer share the same fitness: fitness becomes *context-dependent*. The same happens to 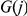, becoming hard to define it analytically. Consequently, we define the ***AGoS*** — denoting it by 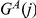 — as the average *i*) over all possible transitions taking place in every node of the network *throughout evolution*, and *ii*) over a large number of networked evolutions (see Methods). This ***AGoS***, which must be computed numerically, becomes therefore network dependent but context independent, as it recovers its population averaged, or mean-field, character. Hence, the ***AGoS*** may constitute a powerful tool to understand dynamical processes at a population-wide scale stemming from individually defined, but often seemingly unrelated, rules.

**Figure 1:**
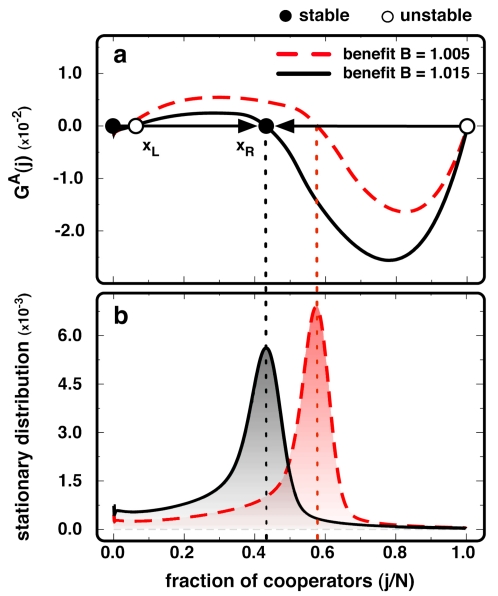
Time-independent AGoS. (**a**) We plot *G^A^(j)* for a population of players interacting via a **PD** in a homogeneous random network, for two values of the benefit **B**. Globally, *G^A^(j)* indicates that the population evolves towards a co-existence scenario. (**b**) Stationary distributions showing the pervasiveness of each fraction *j/N* in time. In line with the ***AGoS*** in **a**), the population spends most of the time in the vicinity of the stable-like root *x_R_* of *G^A^(j)*. When *j/N≈0*, ***C***s become disadvantageous, giving rise to an unstable-like root *x_L_* of *G^A^(j)* which, however, plays a minor role as shown (***N*** *= 10^3^*, *<k> = 4* and β = *1.0*). Homogeneous random networks were obtained by repeatedly swapping the ends of pairs of randomly chosen links of a regular lattice [54].

The results for 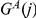 on homogeneous networks of size *N* = 10^3^, *k* = 4 and different values of *B* are shown in Fig. 1a. Unlike well-mixed populations where cooperation has no chance, homogeneous networks can sustain cooperation [12], [14], [15], [24], [54]. The shape of 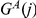 no longer pictures a defection dominance dilemma typical of a **PD**, but a gradient of selection similar to what one observes under co-existence dilemmas in well-mixed populations [43]. In other words, even though every individual engages in a **PD**, from a global, population-wide perspective, homogeneous networks are able to create an emerging collective dynamics promoting the co-existence between ***C***s and ***D***s. As we show below, the emergence of an unanticipated global (macroscopic) dynamics from a distinct individual (microscopic) dynamics pervades throughout all evolutionary dynamical processes in structured populations studied here. The co-existence point *x_R_* (see Fig. 1a) is associated with the internal root (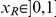) of 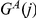
— inexistent in well-mixed populations — whose location decreases with increasing *B*. Together with *x_R_* one obtains a coordination root (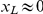, see Fig. 1a) of 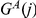 since, in the absence of cooperative partners, ***C***s will always be disadvantageous. However, the impact of 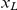 is minor, as shown in Fig. 1b (see discussion below). Remarkably, this characterization remains valid for other types of homogeneous networks, such as lattices and regular rings (as well as for other possible mechanisms of strategy update) whereas differences in the positions of the stable root (*x_R_*) in of 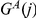 and their dependence on *B* correlate perfectly with results obtained previously [14], [18], [24], [54]–[58], where steady-states of evolution were analyzed for such structures (see Text S1).

Fig. 1a shows that, as we change focus from an individual to a population wide perspective, one witnesses the emergence of an effective game transformation, as evidenced by 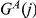, which brings along important consequences: For instance, the fixation time — the time required for cooperators to invade the entire population —becomes much larger in homogeneous networks when compared to well-mixed populations, as the population spends a large period of time in the vicinity of the *x_R_*, mainly when selection is strong (see Methods). This, in turn, is responsible for computer simulations to spend arbitrary amounts of time in the same configuration, even when, in the absence of mutations (as is the case here), the only absorbing states are associated with monomorphic configurations of the population, that is, with configurations comprising cooperators-only or defectors-only. The stationary distribution (Fig. 1b), which represents the pervasiveness of each fraction of ***C***s in time, confirms the scenario portrayed by 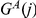 in Fig. 1a, stressing the similarities with the evolutionary dynamics in (finite) well-mixed populations under co-existence dilemmas [50], [59], [60], and putting in evidence the marked difference between individual preferences and the population-wide dynamics.

**Figure 2:**
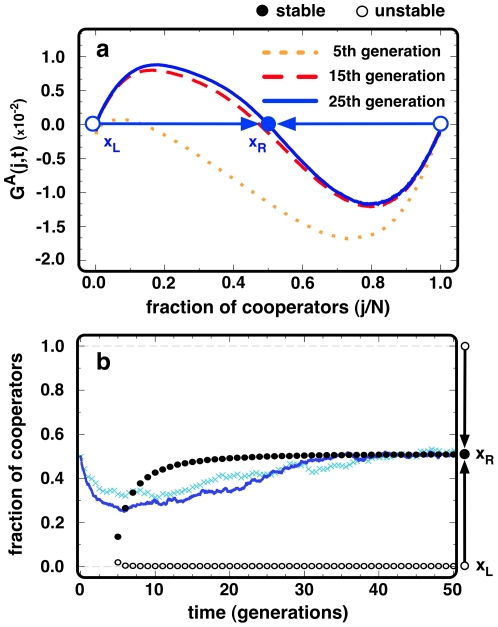
Time-dependent AGoS. (**a**) We plot *G^A^(j,t)* for three different instants of evolutionary time. Each line provides a snapshot for a given moment, portraying the emergence of a population-wide (time-dependent) co-existence-like dilemma stemming from an individual (time-independent) defection dominant dilemma (**PD**). (**b**) The circles show the position of the different interior roots of *G^A^(j,t)*, whereas the solid (dark blue) line and (light blue) crosses show two independent evolutionary runs starting from *50%* of ***C***s and ***D***s randomly placed in the networked population. Open (full) circles stand for unstable, *x_L_* (stable, *x_R_*) roots of *G^A^(j,t)* (***B*** *= 1.01*, ***N*** *= 10^3^*, *<k> = 4* and *β = 10.0*).

In Fig. 1, our analysis was limited to 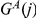 that is, we averaged over the entire time span of all runs. However, the ***AGoS*** itself evolves in time — 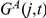
— as detailed in Methods. Let us explore this time dependence of the ***AGoS***. If, at the beginning of each simulated evolution, ***C***s and ***D***s are randomly spread in the network, the occurrence of clusters of the same strategy will not occur in general. Hence, for the **PD** we have that 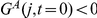 in general. As populations evolve, ***C***s (***D***s) breed ***C***s (***D***s) in their neighborhood, promoting the assortment of strategies, with implications both on the fitness of each player and on the shape (and sign) of 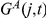. In Fig. 2a we plot 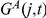 for three particular generations, whereas Fig. 2b portrays the time evolution of the internal roots of 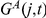, on which we superimposed two evolutionary runs starting with 50% of ***C***s randomly placed in the population. As 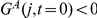, the fraction of cooperators will start decreasing (Fig. 2a). However, with time, strategy assortment leads to the emergence of a co-existence root, toward which the fraction cooperators converges. The ensuing coexistence between ***C***s and ***D***s, entirely described by the shape of 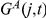, steams from the self-organization of ***C***s and ***D***s in the network, defining a global dynamics which is impossible to foresee solely from the nature of the local (**PD**) interactions.

It is now generally accepted, however, that homogeneous networks provide a simplified picture of real interaction networks [5], [44], [61], [62]. Most social structures share a marked heterogeneity, where a few nodes exhibit a large number of connections, whereas most nodes comprise just a few. The fingerprint of this heterogeneity is provided by the associated degree distributions, which exhibit a broad-scale shape, often resembling a power-law [4], [44], [61]. In the following we use 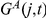 to understand how heterogeneity shifts the internal roots in Fig. 1 to the right, thereby transforming a co-existence scenario into a coordination one. To this end, we compute 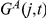 employing scale-free (**SF**) networks of Barabási and Albert (**BA**) (see Methods) [61].

**Figure 3:**
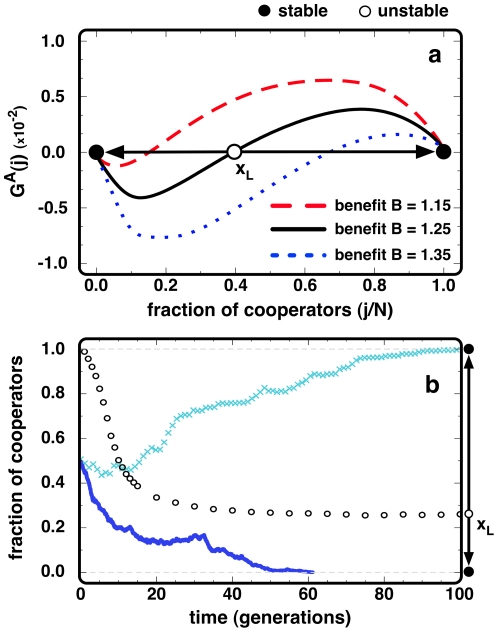
AGoS on BA networks. (**a**) Starting from a defection dominant **PD** played at an individual level, a coordination dynamics emerges at a global, population-wide scale, for the three values of **B** depicted. (**b**) Evolution of the unstable root *x_L_* of *G^A^(j,t)* (open circles), exhibiting the time-dependence of the global dynamics; solid (dark blue) line and (light blue) crosses show two independent evolutionary runs starting from *50%* of ***C***s and ***D***s randomly placed. The ultimate fate of ***C***s in each run depends on whether the population composition crosses over the time-dependent value *x_L_* of *G^A^(j,t)* thereby overcoming the dynamical coordination barrier during evolution. (***B*** *= 1.25*, ***N*** *= 10^3^*, *<k> = 4* and *β = 0.1*). **BA** networks were obtained combining growth and preferential attachment, following the model proposed by Barabási and Albert [61].

Fig. 3a shows 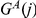 for **BA** networks of *N* = 10^3^ nodes and an average degree *k* = 4, whereas the circles in Fig. 3b portray the time evolution of the internal roots of 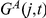. Heterogeneous networks lead to a global dynamics dominated by a coordination threshold, originating the appearance of two basins of attraction split by an unstable root *x_L_* of 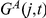, analogous to the evolutionary dynamics under 2-person and N-person Stag-hunt dilemmas in unstructured populations [45], [51], [63], [64]. This unstable root represents the critical fraction of ***C***s above which they are able to assort, thereby invading highly connected nodes, rendering cooperation an advantageous strategy, as ***C***s acquire a higher probability of being imitated than ***D***s. On **SF** networks the requirement to reach the *hubs*, which ensures the formation of cooperative star-like clusters [39], [52], makes invasion harder for isolated ***C***s. This moves the unstable root located close to *j*/*N≈0* in homogeneous networks (see Fig. 1) to higher fractions of ***C***s. Yet, once this coordination is overcome, ***C***s benefit from the strong influence of hubs to rapidly spread in the population, eventually leading to fixation. As a result, the stable internal root which characterizes 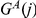 in homogeneous networks collapses into the vicinity of *j = N* on heterogeneous structures, promoting the evolution towards fully cooperative populations. Naturally, the location of the unstable root of 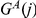 is an increasing function of *B* (see Fig. 3a). It is noteworthy that our results remain qualitatively valid for other update rules, such as the discrete analogue of the replicator dynamics on graphs, used in many references, e.g., [16], [27], [38], [65]. In fact, the ***AGoS*** is capable of identifying particular features of such dynamics: For instance, the partially deterministic nature of such update rule may lead to evolutionary deadlocks in heterogeneous (scale-free) networks, creating stationary states close to full cooperation [16], [27]. In such situation, the ***AGoS*** will reflect the occurrence of these stationary configurations by shifting to the left-hand side the stable (*x_R_*) *equilibrium*, which may no longer coincide with *j = N*, remaining, however, in its vicinity.

The existence of a coordination barrier for ***C***s in heterogeneous networks, which must first occupy the hubs before outcompeting ***D***s, leads to an intricate interplay between the time-dependent decline of *x_L_* (see Fig. 3b) and the global fraction of ***C***s. In Fig. 3b we show, with full lines, two evolutions in **BA** networks (for the same value of *B* = 1.25): One, which fixates in full cooperation and another, which fixates in full defection. Whenever the fraction of cooperators *j/N* remains sizeable for long enough, *x_L_* will eventually decrease to values satisfying *j/N*>*x_L_*, such that the global coordination barrier is overcome and the population will fixate into full cooperation (light blue line). Otherwise, *j/N* may remain always below *x_L_* with the population fixating into full defection (dark blue line). Clearly, heterogeneous networks lead to the emergence of a global coordination barrier and associated basins of attraction that evolve in time, in a way which is well described by the time-dependent ***AGoS***.

## Discussion

To establish the link between individual and collective behavior constitutes, undeniably, one of the main goals of the analysis of any complex multi-particle or multi-component system [66]. Here we establish such a link showing how it depends on the underlying network topology. Our study shows that behavioral dynamics of individuals facing a cooperation dilemma in social networks can be understood as though the network structure is absent but individuals face a different dilemma: The structural organization of a population of self-regarding individuals helps circumventing the Nash equilibrium of a cooperation dilemma by creating a new dynamical system that can be globally characterized by two internal fixed points, *x_L_* (unstable) and *x_R_* (stable). While a single defector will be always advantageous (creating an unstable fixed point at *x* = 1.0), a single cooperator will be always disadvantageous (prompting a stable equilibrium at *x* = 0.0). As cooperators assort into stable clusters, they may also become advantageous above a certain critical fraction of cooperators (*k/N*>*x_L_*, associated with a critical cluster size) and below another critical fraction of cooperators *x_R_*, above which defectors will be able to ripe again the benefits of exploiting the many surrounding cooperators. Whereas in homogeneous networks the stable *equilibria* dictate the overall dynamics — as in co-existence dilemmas — heterogeneous networks create a global dynamics mainly dominated by the unstable *equilibria*, creating a coordination problem.

Strictly speaking, such a dynamical system resulting from individuals interacting (locally) via a two-person game, cannot be mapped onto a two-person evolutionary game in a well-mixed population, since the latter can only comply with a maximum of one internal fixed point [43]. On the contrary, such dynamics resembles that from, e.g., *N*-person dilemmas [67], [68] in the presence of coordination thresholds [51],[64],[69]. It is as if the global dynamics of a 2-person dilemma in structured populations can be properly described as a time-dependent *N*-person dilemma, in which the coordination or co-existence features emerge from the population structure itself, with different network topologies emphasizing differently this co-existence/coordination dichotomy.

It is worth emphasizing that the approach developed here in the context of the two-person **PD** may be useful ― and immediately applicable ― in understanding the evolutionary dynamics of other game interactions, as well as in understanding other aspects of human sociality that extend beyond cooperation. From human behaviors and ideas, to diseases spreading or to individual preferences, most have been modeled as a person-to-person spreading process embedded in a social network [5], [8], [62]. In such frameworks, the identification and categorization of the global, population-wide dynamics which emerges from the apparently unrelated nature of the local interactions may enable one to anticipate the emergent outcomes of such complex biological and social systems.

## Methods

Evolution is modelled via a stochastic birth-death process [47], [70], [71]. Each individual *x* adopts the strategy of a randomly selected neighbour *y* with probability given by the Fermi function 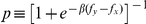 [14], [47], where *f_x_* (*f_y_*) stands for the accumulate payoff of *x* (*y*) and 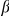 controls the intensity of selection. In structured populations, the difference of the probabilities to increase and decrease the number of ***C***s (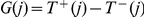) becomes context dependent, but can be computed numerically. For each individual *i* we compute the probability of changing behavior at time *t*, 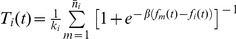, where 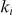 stands for the degree of node *i* and 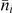 for the number of neighbors of *i* having a strategy different from that of *i*. The *time-dependent* ***AGoS*** at a given time *t* of simulation *p*, where we have *j* ***C***s in the population of size *N*, is defined as 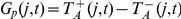, where 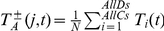. For a given network type, we run Ω = 2×10^7^ simulations (using 10^3^ randomly generated networks) starting from all possible initial fractions *j*/*N* of cooperators. Each configuration of the population is defined here by the fraction *j*/*N* of cooperators. Evolutions run for Λ = 10^5^ time steps. Hence, the overall, *time-independent*, ***AGoS*** is given by the average 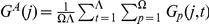 over all simulations and time-steps. The *time-dependent* gradients 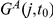 for a particular *generation t*_0_ (and corresponding roots shown in Fig. 2 and 3b) were computed averaging over the configurations occurring during *N* previous time-steps (1 generation): 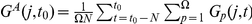. The stationary distributions pictured in Fig. 1b were obtained computing the fraction of time the population spent in each overall configuration *j*/*N*. In some specific limits — in particular, for weak selection or well-mixed populations — our numerical approach will provide results analogous to those obtained with other methods (see for instance [20], [22], [43], [46], [47], [50], [60], [71]–[74]).

Homogeneous random networks were obtained by repeatedly swapping the ends of pairs of randomly chosen links of a regular network [54]. ***BA*** networks were obtained combining growth and preferential attachment, following the model proposed by Barabási and Albert [4], [61]. All networks used have *N* = 10^3^ nodes and an average degree *k* = 4.

## Supporting Information

Text S1 **Supporting Text (containing one additional figure) on the analysis of the evolutionary steady states in homogeneous networks.**
(PDF)
