# Supplementary material for: From Local to Global Dilemmas in Social Networks
Source: PLoS One. 2012 Feb 21;7(2):e32114. doi: 10.1371/journal.pone.0032114 (PMC3283728; doi:10.1371/journal.pone.0032114)
Supplement: Text S1 — Supporting Text (containing one additional figure) on the analysis of the evolutionary steady states in homogeneous networks. (PDF) [file pone.0032114.s001.pdf]

SUPPORTING INFORMATION – TEXT S1

From Local to Global Dilemmas in Social Networks

Flávio L. Pinheiro[1], Jorge M. Pacheco[2,1] and Francisco C. Santos[3,1, ‡]

[1] ATP-group, CMAF, Instituto para a Investigação Interdisciplinar, P-1649-003 Lisboa Codex, Portugal.

[2] Departamento de Matemática e Aplicações, Universidade do Minho, 4710 - 057 Braga, Portugal.

[3] Departamento de Engenharia Informática, Instituto Superior Técnico, Av. Rovisco Pais, 1, 1049-001 Lisboa, Portugal.

[‡] **Corresponding Author**: Francisco C. Santos, ATP-Group, Centro de Matemática e Aplicações Fundamentais, Complexo Interdisciplinar da Universidade de Lisboa, Av. Prof. Gama Pinto, 2,1649-003 Lisboa, Portugal, email: xsantos@gmail.com; tel: +351.21.7904891; fax:+351.21.7954288/89

Evolutionary steady states in homogeneous networks

As we argue in the main text, the shape of the time independent $G^A(j)$ obtained for homogeneous networks indicates that these topologies induce a co-existence game dynamics in a population of individuals engaging in a Prisoner's Dilemma (**PD**). Moreover, the stationary regime is associated with the interior root of $G^A(j)$. Thus, it is reasonable to expect that the stable roots of $G^A(j)$ will coincide with the steady states obtained from computer simulations carried out on the same networks.

In Fig. S1 we compare the interior roots of $G^A(j)$ (circles) with the stationary states (lines) obtained via computer evolutions [1-5] carried out for several values of the benefit ***B*** and for homogenous networks, ranging from ordered lattices (*Lattice*) to random networks (*HoRand*). Fig. S1 confirms that the information offered by $G^A(j)$ remains valid and strikingly accurate for a broad range of game parameters for both types of networks. In accord with the results in the main text, the stationary states were computed for networks with N=1000 individuals and an average connectivity of z=4. As before, each individual revise his or her strategy adopting the one of a randomly selected neighbour with probability given by the Fermi function (see Methods) [2,6]. Each equilibrium fraction of cooperators in a simulation was obtained by averaging over 500 generations after a transient period of 10^4 generations starting from 50% of ***C***s randomly placed on the network. Both red and black lines in Fig. S1 correspond to a subsequent average over 10^4 simulations.

$G^A(j)$ and its interior roots (full circles in Fig. S1) were computed for the same game and network parameters by averaging $G^A(j,t)$ over 100 generations after a transient of 50 generations (see Methods for details of computation of $G^A(j,t)$).

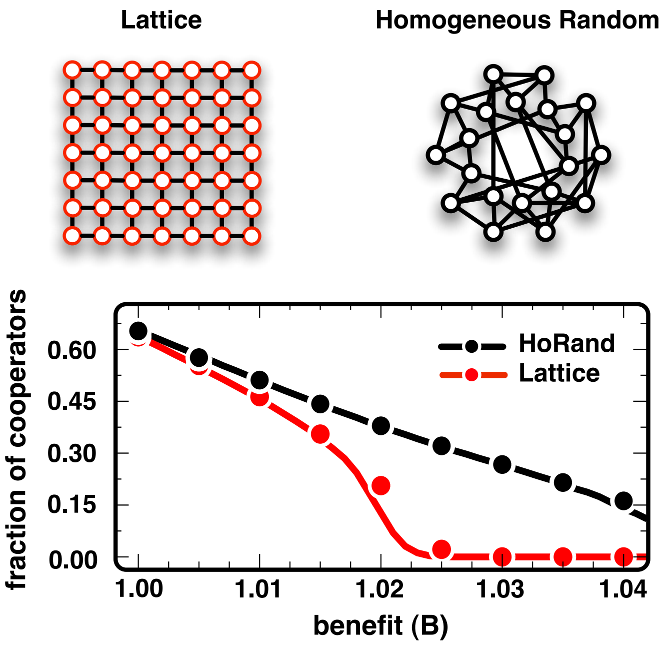

Fig.S1. Evolutionary dynamics cooperation in homogeneous networks. We plot the interior roots x_R of $G^A(j)$ (circles) for a **PD** (*T=B*, *R=1*, *P=0*, *S=1-B*) in homogeneous networks, from random networks (black circles) to ordered lattices (red circles), as a function of the benefit *B*. $G^A(j)$ indicates that the population evolves towards a stationary fraction x_R of ***C***s. This is confirmed by the stationary states (lines) obtained via computer simulations starting from 50% of ***C***s and ***D***s randomly placed in each network. (N=10^3, k=4 and β=0.1).

References

1. Nowak MA, May RM (1992) Evolutionary games and spatial chaos. Nature 359 826-829.
2. Szabó G, Toke C (1998) Evolutionary prisoner's dilemma game on a square lattice. Phys Rev E 58 69-73.
3. Santos FC, Rodrigues JF, Pacheco JM (2005) Epidemic spreading and cooperation dynamics on homogeneous small-world networks. Phys Rev E 72 (5 Pt 2): 056128.
4. Hauert C, Szabó G (2005) Game theory and physics. Am J Phys 73 (5): 405-414.
5. Szabó G, Fáth G (2007) Evolutionary games on graphs. Phys Rep 446 (4-6): 97-216.
6. Traulsen A, Nowak MA, Pacheco JM (2006) Stochastic dynamics of invasion and fixation. Phys Rev E 74 (1 Pt 1): 011909.
